# Management and Leadership Development in Healthcare Professionals

**DOI:** 10.31729/jnma.8100

**Published:** 2023-03-31

**Authors:** Lochan Karki, Badri Rijal, Pawan Kumar Hamal, Milan Chandra Khanal, Suzit Bhusal

**Affiliations:** 1 Nepal Medical Association, Exhibition Road, Kathmandu, Nepal; 2 Journal of Nepal Health Research Council, Ram Shah Path, Kathmandu, Nepal; 3 Journal of Nepal Medical Association, Exhibition Road, Kathmandu, Nepal

**Keywords:** *leadership*, *Nepal*, *public health*, *training activities*

## Abstract

In the healthcare system, leadership has never been more crucial. Initiatives to improve health care in underdeveloped nations frequently fail, not for lack of clinical and public health understanding, but rather for lack of management ability. However, there are currently few possibilities for thorough leadership development at any level of career. This short communication highlights the success of the International Public Health Management Development Program by the Nepal Medical Association in conjunction with the Indian Embassy in Nepal, financed by the Ministry of External Affairs under the Indian Technical Education Corporation.

## INTRODUCTION

The necessity of formal leadership training and its significance in the field of health care have become much more widely recognized during the last 15 to 20 years. It is an important consideration in highly regarded healthcare organizations because capable, excellent executives who exhibit a collective leadership style are necessary to promote excellent patient care.^1,2^ With little expectation of understanding of the so-called distinguishing abilities, such as finances, team building, communication skills, and emotional intelligence, promotion to leadership roles in medicine historically depended on the candidate's academic or clinical accomplishments.

## NEED

The top-down, paternalistic type of leadership, where the leader is completely in charge and demands performance from others, has given way to a more collaborative style, where the leader helps his or her team define a vision and gives them the authority to achieve the stated goals.^3^ Although they might lay the groundwork for future development, early career programs are probably insufficient to develop the leadership abilities required for senior career prospects. To make thorough leadership training more widely available, more development programs are required. A culture of true collaborative or shared leadership would result from improved interactions between hospital administrators, practicing professionals, and academicians as well as improved coordination among disciplines and expanded availability of comprehensive leadership development opportunities. Senior health care leaders would also be better prepared as a result.

Many have advocated for the necessity of formal training in the many facets of leadership, and that it should start at an early career stage.^4-6^ Nonetheless, the number of comprehensive leadership training opportunities at any career level remains limited today.^7-9^

## INTERNATIONAL PUBLIC HEALTH MANAGEMENT DEVELOPMENT PROGRAM

Health care development initiatives in developing countries often fail not due to a lack of clinical and public health knowledge skills but due to a lack of managerial competence. Improvement in healthcare indices requires visionary and innovative approaches from a leader who is skilled to plan, execute and monitor healthcare projects. The Nepalese healthcare system also faces similar problems when it comes to delivering effective healthcare and achieving its healthcare outcomes. The Nepalese medical and public health education system lacks the necessary exposure and ingredients when it comes to developing visionary leaders who have the core managerial competencies to achieve meaningful outcomes.^10^

Realizing this fact, Nepal Medical Association (NMA) takes a step forward to train and improve the exposure of selected future healthcare leaders in collaboration with the Embassy of India in Nepal, sponsored by the Ministry of External Affairs under the Indian Technical Education Corporation (ITEC).^11^ The first batch of the program comprised a mix of senior to middle-level program managers, clinical specialists, academicians and public health professionals who participated in the International Public Health Management Development Program (IPHMDP) organized by the Department of Community Medicine and School of Public Health in Postgraduate Institute of Medical Education and Research (PGIMER), Chandigarh, India where they were exposed to unique opportunities to develop managerial capacity, networking with iconic health care leaders with exceptional health care delivery experiences in solving complex public health issues with sharing of examples of best practices in both the health care system.^12^

The programme aims to build the capacity of middle and senior-level managers in designing, implementing, monitoring and evaluating their program operations in their local context and equip them with management and leadership skills to appreciate the gaps in current health scenarios and envision innovative approaches for effective decision making. The program brings a unique style of teaching methodologies like case studies, gameplay, and cultural events which ultimately help participants learn different strategies to address public health problems in their home countries. The training also offered a unique amalgamation of cultural and study tours to various healthcare facilities along with an intensive scientific program. This helps appreciated future healthcare leaders understand, analyze and network to bring the idea back to their local and national context and improve the health and well-being of their populations.

The officials of the Nepal Medical Association along with 45 healthcare professionals who participated had high praise and feedback for the program and expressed their desire for future collaboration in areas of training, research and development. Key developments were a continuation of the programs, sharing of the human resources for local capacity buildings, and collaborative research and developments. The partnership between the two nations' institutions offers a unique blend of sharing health care issues and helping each other find innovative solutions with the use of knowledge, experiences and technologies.

## WAY FORWARD

The commencement of this program as a part of the leadership and management program highlights the need for further programs. There are data to support the optimal methodology, and opportunities are increasing, although not yet reaching all individuals who might benefit. With resources and expertise, these obstacles may be overcome in a reasonable time. Health care systems, academic institutions, and the practitioners themselves would be well served to find ways to make formal leadership development accessible and part of the routine career evolution for emerging health care leaders.
